# Brg1 Is Required for Cdx2-Mediated Repression of Oct4 Expression in Mouse Blastocysts

**DOI:** 10.1371/journal.pone.0010622

**Published:** 2010-05-12

**Authors:** Kai Wang, Satyaki Sengupta, Luca Magnani, Catherine A. Wilson, R. William Henry, Jason G. Knott

**Affiliations:** 1 Developmental Epigenetics Laboratory, Department of Animal Science, Michigan State University, East Lansing, Michigan, United States of America; 2 Department of Biochemistry and Molecular Biology, Michigan State University, East Lansing, Michigan, United States of America; Cincinnati Children's Research Foundation, United States of America

## Abstract

During blastocyst formation the segregation of the inner cell mass (ICM) and trophectoderm is governed by the mutually antagonistic effects of the transcription factors Oct4 and Cdx2. Evidence indicates that suppression of Oct4 expression in the trophectoderm is mediated by Cdx2. Nonetheless, the underlying epigenetic modifiers required for Cdx2-dependent repression of Oct4 are largely unknown. Here we show that the chromatin remodeling protein Brg1 is required for Cdx2-mediated repression of Oct4 expression in mouse blastocysts. By employing a combination of RNA interference (RNAi) and gene expression analysis we found that both Brg1 Knockdown (KD) and Cdx2 KD blastocysts exhibit widespread expression of Oct4 in the trophectoderm. Interestingly, in Brg1 KD blastocysts and Cdx2 KD blastocysts, the expression of Cdx2 and Brg1 is unchanged, respectively. To address whether Brg1 cooperates with Cdx2 to repress Oct4 transcription in the developing trophectoderm, we utilized preimplantation embryos, trophoblast stem (TS) cells and Cdx2-inducible embryonic stem (ES) cells as model systems. We found that: (1) combined knockdown (KD) of Brg1 and Cdx2 levels in blastocysts resulted in increased levels of Oct4 transcripts compared to KD of Brg1 or Cdx2 alone, (2) endogenous Brg1 co-immunoprecipitated with Cdx2 in TS cell extracts, (3) in blastocysts Brg1 and Cdx2 co-localize in trophectoderm nuclei and (4) in Cdx2-induced ES cells Brg1 and Cdx2 are recruited to the Oct4 promoter. Lastly, to determine how Brg1 may induce epigenetic silencing of the Oct4 gene, we evaluated CpG methylation at the Oct4 promoter in the trophectoderm of Brg1 KD blastocysts. This analysis revealed that Brg1-dependent repression of Oct4 expression is independent of DNA methylation at the blastocyst stage. In toto, these results demonstrate that Brg1 cooperates with Cdx2 to repress Oct4 expression in the developing trophectoderm to ensure normal development.

## Introduction

The first cell-fate decision in the preimplantation embryo, the differentiation of the ICM and trophectoderm, is regulated by the transcription factors Oct4 and Cdx2. Initially, both Oct4 and Cdx2 are widely expressed. However, during blastocyst formation Oct4 expression is restricted to the ICM and Cdx2 expression is confined to the trophectoderm [1], [2]. Evidence indicates that suppression of Oct4 in the trophectoderm is mediated by the inhibitory actions of Cdx2. For example, loss of Cdx2 in early mouse embryos results in developmental arrest around the blastocyst stage and widespread expression of Oct4 in the trophectoderm [2]. Furthermore, forced expression of Cdx2 in embryonic stem (ES) cells induces Oct4 repression via Cdx2 binding to the autoregulatory element (ARE) in the Oct4 promoter resulting in a trophectoderm cell-fate [3]. Collectively, these studies highlight the importance of Cdx2 in repression of Oct4 expression in the developing trophectoderm.

While much has been learned about the sequence of morphological and molecular events that lead up to segregation of the ICM and trophectoderm lineages [2], [4]–[7], less is known about the epigenetic processes that facilitate Oct4 repression in the blastocyst trophectoderm. Brahma related gene 1 (Brg1)*-*dependent chromatin remodeling complexes represent a subclass of SWItch/Sucrose NonFermentable (SWI/SNF) ATP-dependent remodelers that play key roles in embryo development and cellular differentiation [8]–[10]. In the nucleus Brg1 and Brg1 associated factors (BAFs) are recruited to target gene promoters via tissue-specific transcription factors to regulate transcription [11]. Previously, we identified an important role for Brg1 in blastocyst development and ES cell pluripotency [9]. RNA interference (RNAi)-mediated knockdown (KD) of Brg1 in early mouse embryos results in developmental arrest at the blastocyst stage, defects in the trophectoderm, and failure to repress Oct4 and Nanog transcription [9]. Furthermore, utilizing genome-wide location analysis we and others showed that Oct4 and Nanog are direct targets of Brg1 in ES cells [9], [12]. Consistent with these findings Brg1 and BAF155 are required for the repression of pluripotency genes in differentiating ES cells [13]. Altogether, these findings suggest that Brg1 plays a critical role around the blastocyst stage when the first cellular lineages are established.

Here we report that Brg1 is an essential co-repressor required for Cdx2-mediated silencing of Oct4 expression in the trophectoderm. We found that Brg1 and Cdx2 interact at the chromatin level to repress Oct4 transcription in blastocysts. These findings point to a novel role for Brg1 in transcriptional regulation of key Cdx2 target genes in the developing trophectoderm to ensure normal embryo development.

## Results and Discussion

### Brg1 regulates Oct4 expression in a stage-specific manner during blastocyst formation

**Figure 1:**
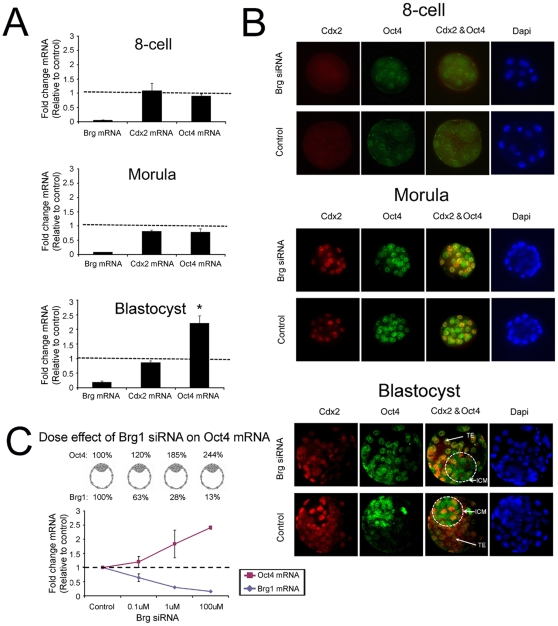
Brg1 is required for repression of Oct4 expression at the blastocyst stage. (A) qRT-PCR analysis of Brg1, Cdx2, and Oct4 transcripts in Brg1 KD 8-cell embryos, morulae, and blastocysts. Data were normalized to Ubtf (house keeping gene) and are relative to control embryos at each stage; black line = 1. Asterisk denotes significant difference between Brg1 KD and control blastocysts (p<0.05). (B) ICC analysis of Oct4 and Cdx2 expression in Brg1 KD 8-cell embryos, morulae, and blastocysts. Nuclei were counter stained with DAPI (blue). (C) Brg1 represses Oct4 expression in a dose dependent manner. One-cell embryos were injected with 0 µM (control), 0.1 µM, 1 µM, or 100 µM Brg1 siRNA and cultured to the blastocyst stage. Real-time qPCR was used to analyze the levels of Brg1 and Oct4 transcripts. Data were normalized to Ubtf and are relative to control blastocysts; dashed line = 1.

Recently, we detected higher amounts of Oct4 transcripts in Brg1 depleted blastocysts compared to control blastocysts [9]. To further assess the potential role of Brg1 in Oct4 regulation, we examined the temporal and spatial expression of Oct4 during blastocyst formation. In addition, we evaluated the expression of the homeobox gene Cdx2, a negative regulator of Oct4 transcription in mouse blastocysts [2], [3]. To accomplish this we microinjected fertilized 1-cell embryos with Brg1 siRNA or control siRNA and cultured them to the 8-cell, morula, and blastocyst stages. The siRNAs utilized in this study are the same siRNAs described previously [9]; they induce specific ablation of Brg1 transcripts in mouse embryos and phenocopy Brg1 null embryos [8]. A combination of quantitative real-time PCR (qRT-PCR) and immunocytochemistry (ICC) was used to examine the expression of Oct4 and Cdx2. At the 8-cell and morula stages we did not detect any differences in the expression or localization of Oct4 and Cdx2 in Brg1 knockdown (KD) versus control embryos (Figure 1A and B; p>0.05). In contrast, at the blastocyst stage, we observed a significant increase in Oct4 mRNA (Figure 1A; p<0.05) and widespread expression of Oct4 protein in Brg1 KD embryos versus control embryos (Figure 1B). Interestingly, the levels of Cdx2 transcripts in Brg1 KD and control blastocysts were similar (Figure 1A; p>0.05), suggesting that the Cdx2 gene itself is not a transcriptional target of Brg1 in blastocysts.

To further explore the relationship between Brg1 and Oct4 transcription we microinjected various concentrations of Brg1 siRNA (0, 0.1, 1.0, 100 µM) into 1-cell embryos to generate a series of day 4 blastocysts with different amounts of Brg1. We reasoned that if Brg1 is a direct repressor of the Oct4 gene there should be a dose dependency of Brg1 siRNA on Oct4 mRNA expression. qRT-PCR was used to measure the levels of Brg1 and Oct4 transcripts in Brg1 KD blastocysts. Interestingly, we observed an inverse relationship between Brg1 levels and Oct4 expression (Figure 1C). As the levels of Brg1 decreased there was a steady increase in Oct4 mRNA. Altogether, these findings indicate that Brg1 regulates Oct4 expression in a dose dependent manner and is critical for regulation of Oct4 transcription at the blastocyst stage.

### Brg1 KD blastocysts have normal levels of Cdx2 protein and increased amounts of Oct4 protein in the trophectoderm

**Figure 2:**
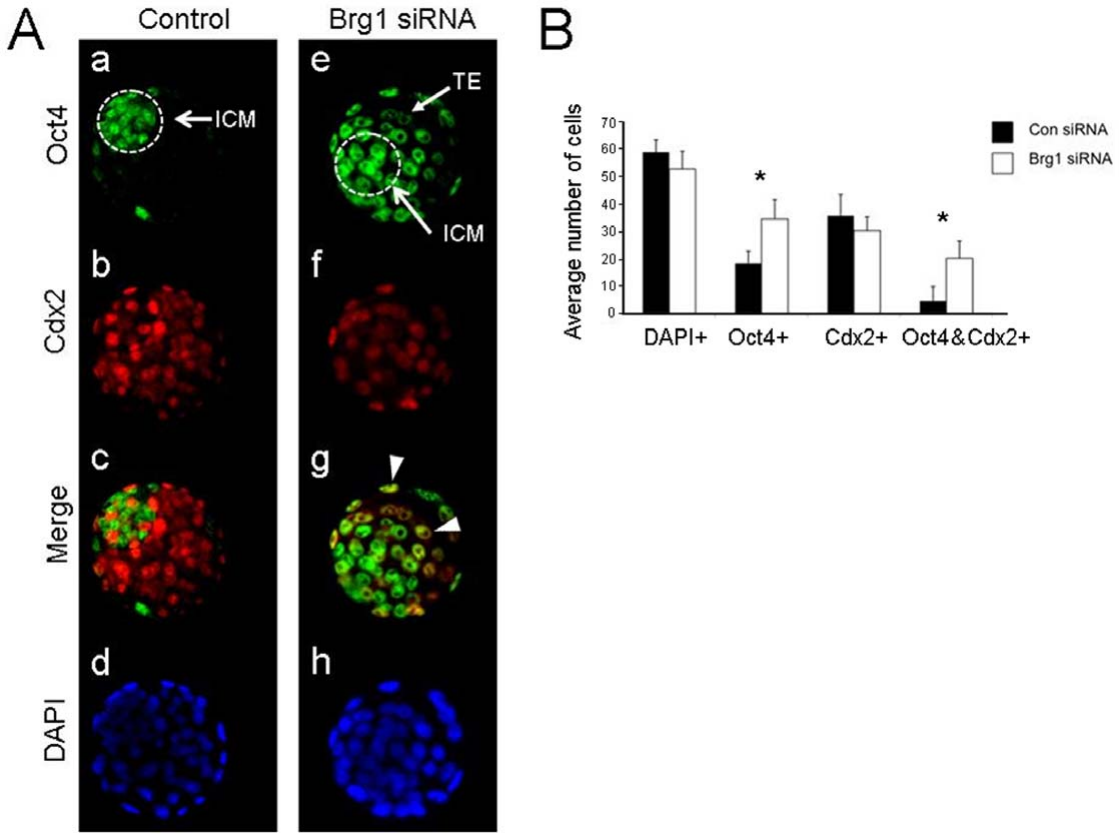
Expression and localization of Cdx2 and Oct4 in Brg1 KD blastocysts. (A) ICC analysis of Oct4 and Cdx2 in Brg1 KD and control blastocysts. In control blastocysts (a–d) Oct4 expression (green) is restricted to the ICM and is largely absent in the Cdx2-positive (red) trophectoderm. In contrast, in Brg1 KD blastocysts (e–h) Oct4 is widely expressed in both the ICM and cdx2-positive (yellow) trophectoderm. Arrowheads denote co-expression of Oct4 and Cdx2. Nuclei were counter stained with DAPI (blue). (B) Quantification of the average number of cells in Brg1 KD and control blastocysts expressing Oct4, Cdx2, and Oct4 & Cdx2 (double expression). Asterisks denote statistical significance (p<0.05) between Brg1 KD and control blastocysts. A total of 25 Brg1 KD blastocysts and 15 control blastocysts were analyzed.

Since Cdx2 is a direct repressor of Oct4 transcription in the trophectoderm [3], we examined the precise expression and localization of Cdx2 and Oct4 in Brg1 KD blastocysts. We reasoned that changes in Cdx2 expression *per se* could be accountable for misexpression of Oct4 in the trophectoderm. Using confocal microscopy we calculated the average number of Oct4+ (green), Cdx2+ (red), and Oct4 & Cdx2+ cells (yellow) in control blastocysts and Brg1 KD blastocysts. In control blastocysts Oct4 expression was restricted to cells in the ICM and was largely absent in the Cdx2+ trophectoderm cells (Figure 2A a–d; Fig. S1). In contrast, in Brg1 KD blastocysts Oct4 was widely expressed in the Cdx2+ trophectoderm (Figure 2A e–h; Fig. S1). Remarkably, there was no difference in the number of Cdx2+ cells (Figure 2B; 30±1.6 vs. 32±4.3; p>0.05) nor the total cell number (Figure 2B; 53±2.0 vs. 59±1.2; p>0.05) between Brg1 KD and control blastocysts. On the other hand, there were approximately twice as many Oct4+ cells in Brg1 KD blastocysts compared to control blastocysts (Figure 2B; 35±1.7 vs. 18±1.9; p<0.05). Most importantly, there were a higher number of cells that co-expressed Oct4 and Cdx2 in Brg1 KD blastocysts versus control blastocysts (Figure 2B; 20±1.9 vs. 4±0.6; p<0.05). Collectively, these results demonstrate that in Brg1 KD blastocysts Oct4 is widely expressed in the trophectoderm and that this phenomenon is not caused by alterations in Cdx2.

### Brg1 cooperates with Cdx2 to repress Oct4 transcription in blastocysts

The phenotype of Brg1 KD blastocysts resembled the phenotype previously described for Cdx2 knockout blastocysts [2]. Moreover, the phenotype of Brg1 KD blastocysts is similar to Cdx2 KD blastocysts that were generated via microinjection of Cdx2 siRNA into one-cell embryos (Figure S2). For example, both Brg1 and Cdx2 KD embryos arrest around the blastocyst stage, exhibit defects in the trophectoderm, and have increased expression of Oct4 in the trophectoderm cells. Interestingly, in Cdx2 KD blastocysts the levels of Brg1 mRNA and protein are similar to control blastocysts further demonstrating that Brg1 and Cdx2 do not regulate each other, but may act together to repress Oct4 transcription (Figure S2). Thus, we hypothesized that Brg1 cooperates with Cdx2 to repress Oct4 transcription in the trophectoderm.

**Figure 3:**
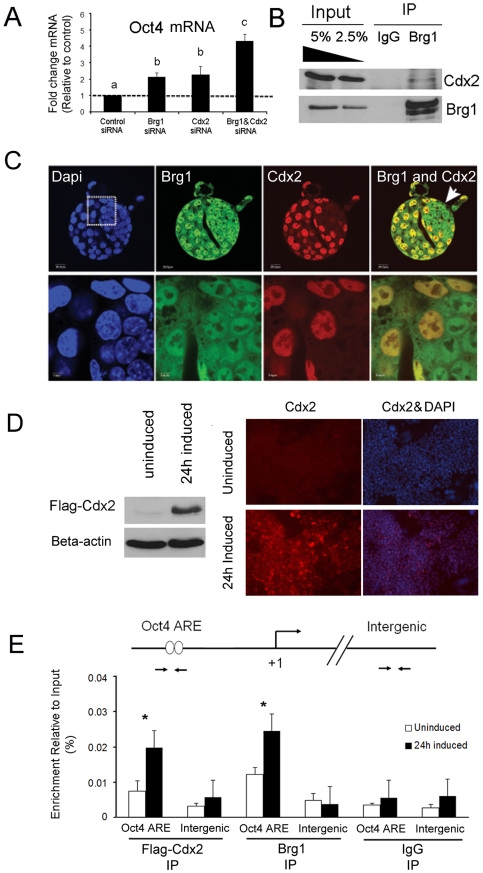
Brg1 cooperates with Cdx2 to repress Oct4 transcription in blastocysts. (A) Combined depletion of Brg1 and Cdx2 augments Oct4 expression in blastocysts. qRT-PCR analysis of Oct4 transcripts in Brg1 KD blastocysts, Cdx2 KD blastocysts, and Brg1 & Cdx2 double KD blastocysts. Data were normalized to Ubtf (house keeping gene) and are relative to control blastocysts; dashed line = 1. Different letters denote statistical significance in Oct4 transcripts (p<0.05). These experiments were replicated using a total of 5 biological replicates. (B) Co-immunoprecipitation and western blot analysis of Brg1 and Cdx2 in TS cells. Brg1 was immunoprecipitated using a rabbit anti-serum. Recovery of Cdx2 was measured by western blot analysis. Cdx2 is enriched in the Brg1 IP samples and not in the control IgG samples. This assay was repeated a total of 4 times using different batches of TS cells. (C) Confocal immunofluorescence analysis of Brg1 and Cdx2 in blastocysts. Co-localization of endogenous Brg1 and Cdx2 in trophectoderm nuclei was determined using specific antibodies for Brg1 and Cdx2. Nuclei were counterstained with DAPI. White box represents magnified region in bottom panel. Arrow denotes blastocyst ICM. (D) Confirmation of Flag-Cdx2 expression in induced ES cells. Western blot and immunofluorescence analysis of Flag-Cdx2 expression at 24 hours following removal of doxycycline. (E) ChIP analysis of Brg1 and Cdx2 binding to the Oct4 promoter in Cdx2-inducible ES cells. qRT-PCR was used to determine the relative enrichment of Brg1 and Flag-Cdx2 at the Oct4 ARE versus an intergenic region in uninduced and induced ES cell extracts. A non-specific rabbit IgG was included as a negative control. Data were normalized to 1% input DNA. Asterisks denote significant differences between uninduced and induced samples (p<0.05). These experiments were replicated 3 to 4 times using two different batches of Cdx2-inducible ES cell extracts.

To test this hypothesis we first examined the effect of combined depletion of Brg1 and Cdx2 levels on Oct4 expression in blastocysts. We predicted that loss of the Brg1-Cdx2 interaction would de-repress Oct4 mRNA expression in blastocysts resulting in higher levels of Oct4 transcripts. Accordingly, 1-cell embryos were either microinjected with Brg1 siRNA (group 1), Cdx2 siRNA (group 2), Brg1 siRNA and Cdx2 siRNA (group 3), or control siRNA (group 4) and cultured to the blastocyst stage. Microinjection of Brg1 siRNA, Cdx2 siRNA, or a combination of both induced a similar decrease in Brg1 and Cdx2 transcripts (Figure S3). Microinjection of Brg1 siRNA or Cdx2 siRNA alone resulted in a 2.1±0.2 and 2.3±0.5 fold increase in Oct4 mRNA compared to control blastocysts, respectively (Figure 3A; p<0.05). Remarkably, in Brg1 & Cdx2 double KD blastocysts we observed a 4.3±0.4 fold increase in Oct4 mRNA compared to control blastocysts (Figure 3A; p<0.05). This increase was significantly greater than the levels of Oct4 in Brg1 KD and Cdx2 KD blastocysts alone (Figure 3A; p<0.05). These results suggest that Cdx2 and Brg1 may function additively to repress Oct4 transcription in blastocysts.

To determine whether Brg1 and Cdx2 associate during trophectoderm development, three sets of experiments were carried out. We first conducted co-immunoprecipitation assays using mouse TS cells as a reference system for the blastocyst trophectoderm. In these experiments, endogenous Brg1 was recovered using a Brg1 rabbit anti-serum [14] and endogenous Cdx2 recovery was measured by Western blot analysis. As shown in Figure 3B, Cdx2 was indeed enriched in samples recovered from the anti-Brg1 immunoprecipitation relative to reactions performed using a rabbit IgG.

To further examine whether Brg1 and Cdx2 associate in the trophectoderm confocal immunofluorescence analysis was carried out in blastocysts using Brg1 and Cdx2 antibodies. This analysis revealed that endogenous Brg1 and Cdx2 co-localize within similar nuclear foci in trophectoderm cells (Figure 3C).

Lastly, we used chromatin-immunoprecipitation (ChIP) analysis to determine whether Brg1 and Cdx2 are recruited to the Oct4 promoter during trophectoderm formation. To accomplish this we utilized a doxycycline controllable Cdx2-inducible ES cell line as a model system for the developing blastocyst trophectoderm [15]; these ES cells transdifferentiate into TS cells following induction of Cdx2 expression. Previously, Rossant and co-workers demonstrated that Cdx2 is recruited to the ARE of the Oct4 promoter within 24 hours after induction of Cdx2 expression [3]. In preliminary experiments we confirmed by Western blot and immunofluorescence analysis that Flag-Cdx2 was strongly induced at 24 hours following removal doxycycline (Figure 3D). Moreover, at 24 to 48 hours after induction Oct4 expression was significantly down-regulated in these cells (data not shown). Remarkably, ChIP analysis revealed that both Brg1 and Cdx2 are recruited to the Oct4 ARE at 24 hours following induction of Cdx2 expression (Figure 3E; p<0.05). The recruitment of Brg1 and Cdx2 to the ARE corresponded to when Oct4 became repressed in these cells. Importantly, enrichment of Brg1 and Cdx2 was not observed at a negative control intergenic region (Figure 3E; p>0.05). Collectively, these results strongly suggest that Brg1 and Cdx2 cooperate at the chromatin level to repress Oct4 transcription during trophectoderm development.

### Brg1-dependent repression of Oct4 expression does not require DNA methylation

**Figure 4:**
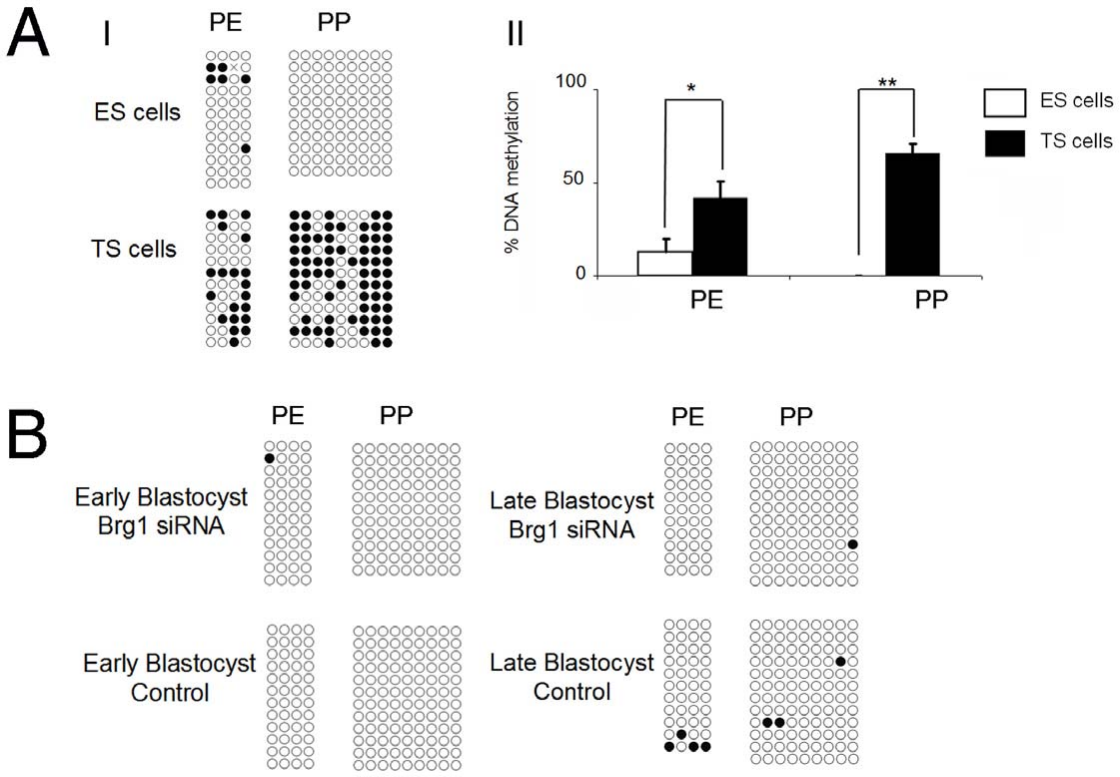
Brg1-dependent repression of Oct4 expression in the trophectoderm does not require DNA methylation. (A I–II) Bisulfite sequencing analysis of the Oct4 proximal enhancer (PE) and proximal promoter (PP) in ES cells and TS cells. Closed circles denote CpG methylation. Asterisks denote statistical significance (p<0.05). (B) Bisulfite sequencing analysis of the Oct4 PE and PP in the trophectoderm of Brg1 KD blastocysts and control blastocysts.

It has been established that Oct4 silencing in TS cells is controlled by epigenetic modifications of chromatin, including DNA methylation [16]. In some cellular contexts, Brg1 represses transcription through recruitment of DNA methyltransferases (DNMTs) to target gene promoters [17]. Thus, we hypothesized that Brg1-mediated repression of the Oct4 expression in blastocysts might involve epigenetic modification events. We decided to test (1) whether the Oct4 gene is repressed via DNA methylation at the blastocyst stage and (2) if disruption of Brg1 influences the methylation status of the Oct4 promoter. To accomplish this we conducted bisulfite-sequencing analysis of genomic regions within the Oct4 proximal enhancer (PE) and proximal promoter (PP) in blastocysts, TS cells, and ES cells. Accordingly, in ES cells the Oct4 PE and PP regions were largely unmethylated consistent with the high levels of Oct4 expression in these cells (Figure 4A I and II). In contrast, in TS cells where Oct4 expression is silenced, the Oct4 promoter was highly methylated in both the PE and PP regions (Figure 4A I and II;P<0.05). We next analyzed the methylation profiles of the trophectoderm of Brg1 KD blastocysts versus control blastocysts. To achieve this we separated the trophectoderm from early blastocysts (day 4.0) and late blastocysts (day 4.5) using laser-mediated dissection and processed them for bisulfite sequencing. First, to confirm that our assay was sensitive enough for smaller pools of embryos we analyzed the differentially methylated region (DMR) of the imprinted Snrpn gene. We found that this region was hemi-methylated in these embryos (data not shown). Interestingly, in the trophectoderm of control blastocysts, irrespective of the stage, the Oct4 PE and PP regions were almost completely unmethylated (Figure 4B). Moreover, in Brg1 KD embryos we did not observe any changes in DNA methylation in the trophectoderm of early or late blastocysts (Figure 4B). No differences in methylation were observed between control blastocysts and Brg1 KD blastocysts (p>0.05). Collectively, these results suggest that Brg1-mediated repression of Oct4 in blastocysts does not require DNA methylation and that other mechanisms are likely important for facilitating Oct4 repression during blastocyst formation.

### A model for Brg1/Cdx2-mediated repression of Oct4 expression in trophectoderm

**Figure 5:**
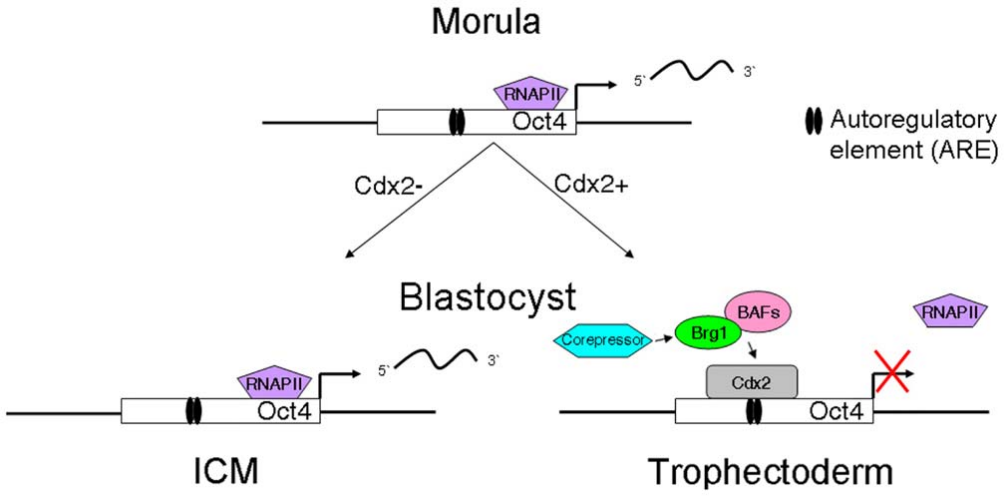
Model for Brg1/Cdx2-mediated repression of Oct4 expression in trophectoderm. Schematic diagram of Oct4 regulation in blastocysts. In the trophectoderm Brg1 is recruited to the ARE of the Oct4 promoter via Cdx2. Once at the Oct4 promoter Brg1 and Cdx2 facilitate recruitment of additional co-repressors to repress transcription.

We describe herein a novel role for Brg1 and Cdx2 in regulation of Oct4 expression in blastocysts. To date little is known about the repressive function of Brg1 during blastocyst formation and establishment of the first cellular lineages. Moreover, the underlying epigenetic processes responsible for Cdx2-mediated repression of Oct4 in the trophectoderm are largely unknown. Our results in preimplantation embryos and TS cells show that: (I) Brg1 is required for repression of Oct4 expression in the trophectoderm, (II) Brg1 cooperates with Cdx2 to repress Oct4 transcription, and (III) Brg1 does not require DNMT activity to repress Oct4 expression at the blastocyst stage. Therefore, we propose a two-step model to explain the repression of Oct4 transcription in the blastocyst trophectoderm. Firstly, we predict that Brg1 and Cdx2 are recruited to the ARE of the Oct4 promoter during blastocyst formation (Figure 5). This view is supported by our findings in Cdx2-inducible ES cells where we observed a significant enrichment of both Cdx2 and Brg1 at the Oct4 promoter 24 hours after induction of Cdx2. The temporal order in which Brg1 and Cdx2 are recruited to the Oct4 promoter is currently not known. Notably, in some cell-types Brg1 is recruited to target gene promoters via tissue-specific transcription factors [11], [14]. We envision that a similar mechanism may exist in blastocysts. In future experiments we will determine whether the recruitment of Brg1 to the ARE of the Oct4 promoter depends on Cdx2.

Secondly, we theorize that once at the Oct4 promoter, Brg1 and Cdx2 cooperate to facilitate chromatin remodeling and/or targeting of other co-repressors to induce Oct4 repression (Figure 5). In some cellular contexts Brg1 can associate with HDACs and DNMTs to repress transcription [17], [18]. In the current study we addressed whether DNA methylation was required for Brg1-dependent repression of Oct4 expression in blastocysts. Interestingly, DNA methylation does not appear to be required for the initial repression of Oct4 expression during blastocyst formation. This finding is consistent with another study that showed that the Nanog promoter is largely unmethylated in blastocysts around the time Nanog becomes restricted to the ICM [19]. Conversely, in TS cells Oct4 and Nanog silencing is tightly associated with DNA methylation [16], [ 20, and this study]. This suggests that the silencing of Oct4 gene in the trophectoderm lineage may be facilitated by a series of sequential epigenetic modifications that initiate early on during blastocyst formation and ensue during blastocyst development. We hypothesize that histone modifications such as histone deacetylation are required for the initial repression of Oct4 during blastocyst formation. In support of this notion immunoprecipitation of Cdx2 in Cdx2-inducible ES cell extracts resulted in a significant enrichment of HDAC1/2 [15]. Future studies will address whether Brg1 and/or Cdx2 are necessary for the recruitment of HDAC1/2 to the Oct4 promoter during the early stages of trophectoderm development.

In conclusion, Brg1 is obligatory for Cdx2-mediated repression of Oct4 expression in blastocysts. It is likely that Brg1 is critical for transcriptional regulation of other Cdx2 target genes in the developing embryo. Our results provide a foundation for further examination of these mechanisms.

## Materials and Methods

### Superovulation, embryo collection, microinjection, embryo culture, and outgrowth analysis

B6D2/F1 female mice aged 6–8 weeks (Jackson Laboratory, Bar Harbor, ME) were superovulated as previously described [9] and mated with B6D2/F1 males. Fertilized one-cell embryos were collected in M2 medium (Sigma-Aldrich, St. Louis, MO), washed, and cultured in potassium simplex optimized medium (KSOM) with amino acids (Specialty Media, Phillipsburg, NJ). Microinjections were carried out as described previously [21]. In brief, 5–10 pL of 100 µM Brg1, Cdx2, or control siRNA (siGenome; Dharmacon, Inc., Lafayette, CO) was injected into the cytoplasm of one-cell embryos using a PL100 picoinjector (Harvard Apparatus, Hollistan, MA). Following injection, embryos were cultured in KSOM for 2 to 4 days depending on the experiment. Outgrowth analysis was carried out on day 4 blastocysts by removing zona pellucidae with acid Tyrode (Sigma-Aldrich), washing in M2 medium, and culturing in Dulbecco's modified Eagle's medium (DMEM) supplemented with 15% fetal bovine serum (FBS) and 1,000 U/ml of leukemia inhibitory factor (LIF). After 96 hours the percentage of blastocysts that attached and underwent outgrowth was calculated. All animals were treated in accordance with Institution Animal Care and Use Committee guidelines under current approved protocols at Michigan State University.

### Embryonic stem (ES) cell and trophoblast stem (TS) cell culture

R1 ES cells were, obtained from American Type Culture Collection (ATCC, Manassas, VA), were cultured on mitomycin-treated mouse embryonic fibroblasts (MEFs) in medium containing high-glucose DMEM supplemented with fetal calf serum (FCS), LIF, L-glutamine, nonessential amino acids, β-mercaptoethanol. Cdx2-inducible ES cells were kindly provided by Dr. Minoru Ko [15]. These cells were cultured on mitomycin-treated puromycin resistant MEFs in ES cell medium supplemented with 0.2 µg/ml of doxycycline and 1.0 µg/ml of puromycin. Prior to Cdx2-induction cells were switched onto gelatin and cultured in the presence of 1.5 µg/ml of puromycin for 3 days. Transgene expression was induced by removal of doxycycline and was verified by Western blot using a Flag antibody (F3165; Sigma- Aldrich). Cdx2 expression was confirmed by immunocytochemistry using a Cdx2 antibody (CDX2-88; Biogenex, San Ramon, CA). TS cells were derived from day 4 blastocysts as described by Rossant and co-workers [22]. TS cells were cultured on mitomycin-treated MEFs in RPMI medium containing fetal bovine serum (FBS), sodium pyruvate, L-glutamine, β-mercaptoethanol, fibroblast growth factor 4(FGF4), and Heparin. Two to 3 days before harvesting for biochemical assays they were switched onto gelatin coated plates, minimizing contamination by MEFs.

### RNA isolation, cDNA synthesis, and real-time qPCR analysis of preimplantation embyros

Brg1 KD and control embryos were transferred into a 1.5-mL tube in ∼1 µL of M2 medium and immediately stored at −80°C until used. Total RNA was isolated using the PicoPure RNA isolation Kit (Molecular Devices, Sunnyvale, CA). Prior to RNA isolation, 2 ng of RNA encoding GFP was added to each sample to act as a carrier. Isolated RNA was then subjected to a single round of cDNA synthesis, and final volume was adjusted so that 1 µL was equivalent to an embryo. qRT-PCR analysis was carried out as described previously [9] using TaqMan probes and an ABI 7500 thermocycler (Applied Biosystems, Foster City, CA). All qRT-PCR experiments were carried out using a total of 3 to 6 biological replicates.

### Immunocytochemistry (ICC)

ICC analysis of preimplantation embryos and Cdx2-Inducible ES cells was performed as previously described, with slight modifications [9]. Briefly, embryos or stem cells were fixed, permeabilized, washed, blocked, and incubated in a 1/100 dilution of antibodies for Oct4 (ab19857; abcam, Cambridge, MA), Cdx2 (CDX2-88; Biogenex), or Brg1 [14] overnight at 4°C. The following day embryos or stem cells were washed 3 times in block solution. For secondary detection, samples were incubated in a 1/1,000 dilution of Alexa Fluor 488 and 594 (Molecular Probes, Eugene, OR), washed, mounted in Vectashield (Vector Laboratories, Burlingame, CA) containing 4,6-diamidino-2-phenylindole, and imaged using a spinning disc confocal module (CARV; Atto Bioscience, Rockville, MD) with Metamorph software.

### Co-immunoprecipitation and Western blot analysis

For immunoprecipitation of Brg1, approximately 2 mg of TS cell lysate (200 µl) was diluted to 500 µl in lysis buffer (50 mM HEPES,150 mM NaCl, 1 mM EDTA, 2.5 mM EGTA, 10% glycerol, 0.1% tween 20) containing protease inhibitors and incubated with 12 µl of Brg1 rabbit anti-serum [14] or equivalent amount of rabbit IgG overnight at 4°C. Following overnight incubation stable complexes were affinity purified by incubation with 50 µl of Protein-G Fast Flow agarose beads (Millipore) for 4 hours at 4°C. Beads bound to immunoprecipitated complexes were washed once in lysis buffer and twice in PBS. Bound proteins were eluted from the beads by boiling in 2X Laemmli buffer and size fractionated using 12.5% SDS-PAGE. CDX2 was detected by Western blot analysis using an affinity purified rabbit anti-CDX2 antibody (A300-692A, Bethyl Laboratories, Inc). In order to avoid intervening signal from immunoglobulin heavy and light chains, HRP-conjugated Protein A (Zymed, Invitrogen, Carlsbad, CA) was used for Western blot detection instead of HRP-conjugated secondary antibody [23].

### Chromatin-immunoprecipitation (ChIP) analysis

Cdx2-inducible ES cells were harvested and chemically crosslinked with 1% formaldehyde (Sigma-Aldrich) for 19 hours at 4°C. We previously determined that these fixation conditions work excellent for Brg1 ChIP [9]. Cells were pelleted, washed with PBS and flash frozen in liquid nitrogen. Pellets were resuspended in ChIP lysis buffer. Cells were sonicated using a Branson Sonifier 450D (Branson, Danbury, CT, http://www.sonifer.com) at 50% amplitude, with 6 1-minute pulses in ice water. Postsonication, samples were centrifuged and flash frozen in liquid nitrogen. Sonicated cell extracts equivalent to 2.5×10^6^ cells were used in subsequent immunoprecipitations. Samples were precleared with protein G Dynabeads (Dynal Biotech, Carlsbad, CA, http://www.invitrogen.com/dynal) in 1 ml of dilution buffer. Cell extracts were incubated overnight at 4°C with 5 µL of Brg1 rabbit anti-serum [14], 2 µg of rabbit anti-Flag (F7425, Sigma-Aldrich), or 2 µg of rabbit non-specific IgG (Millipore). Chromatin antibody complexes were isolated with 50 µL of protein G Dynabeads and washed one time with low-salt buffer, one time with high-salt buffer, one time with LiCl wash buffer, and twice with TE buffer. Protein/DNA complexes were eluted from the beads at 65°C with occasional vortexing. Crosslinking was reversed by addition of NaCl and incubation overnight at 65°C. Extracts were then treated with RNase A and proteinase K, and DNA was purified using an Upstate EZ ChIP kit (Millipore). qRT-PCR was performed on Brg1 ChIP DNA, Flag-Cdx2 ChIP DNA, Input DNA and IgG control DNA using SYBR Green Master Mix reagents with an ABI 7500 sequence detection system. The following primer pair was used to analyze the Oct4 ARE region: forward 5′-TGAACTGTGGTGGAGAGTGC-3′ and reverse 5′-AGGAAGGGCTAGGACGAGAG-3′. Negative control primers for an intergenic region were the following: forward 5′-TTTTCAGTTCACACATATAAAGCAGA-3′ and reverse 5′-TGTTGTTGTTGTTGCTTCACTG-3′.

### Bisulfite-sequencing analysis of DNA methylation

The trophectoderm of day 4 and day 4.5 blastocysts (control and Brg1 KD) was isolated by laser dissection using a 40X laser objective lens and controller (Hamilton Throne Bioscience, Beverly, MA). A total of 10 to 15 isolated trophectoderm from each time point were pooled (∼400 cells), and stored at −80°C for future use. A total of 5 to 7 ES cell or TS cell colonies were isolated, and stored at −80°C. Extraction of genomic DNA and bisulfite mutagenesis sequencing analysis were conducted using the ReadyAmp Genomic Kit (Promega, Madison, WI) and the EZ DNA Methylation Kit (Zymo Research, Orange, CA), respectively, according to the manufacturer's instructions. After bisulfite mutation, DNA was eluted in 20 µL of elution buffer, and subjected 2 successive rounds of PCR amplification (35 cycles each) using primer pairs for the Oct4 Promoter. For the proximal enhancer we used the following outer and inner primer pairs: outer forward primer, 5′-TTTGTAGATAGGTATTTTGAGGGT-3′ and outer reverse primer, 5′-ACAAAACTTCCCCAACTCTCCACC-3′; inner forward primer, 5′-GGGATTTTTAGATTGGGTTTAGAAA-3′ and inner reverse primer, 5′- CTCCTCAAAAACAAAACCTCAAATA-3′. For the proximal promoter we used an outer forward primer, 5′-GGTTTTTAGGTGGGTTTGGAATC-3′ and outer reverse primer, 5′-CAACCAAATCCCTTCACTTACCT-3′; inner forward primer, 5′-AGAGGTATTGGGGATTTTTTTATGT-3′ and inner reverse primer, ′5-AAAATTAATTCCACCTTCTCCAACT-3′. PCR products were verified by running on a 2% agarose gel. Then, PCR products were ligated into the pTOPO 10 vector system (Invitrogen) and 10 to 12 clones were randomly picked for sequencing. A total of two biological replicates were analyzed.

### Statistical analysis

Data from qRT-PCR and DNA methylation experiments were analyzed by SAS software (version 9.0, SAS Institute Inc., Cary, NC). A student's *t*-test was used to determine statistical differences between groups. A p-value of <0.05 was considered significant.

## Supporting Information

Figure S1 Expression and localization of Cdx2 and Oct4 in Brg1 KD and control blastocysts. (A) In control blastocysts Oct4 expression (green) is restricted to the ICM and is largely absent in the Cdx2-positive (red) trophectoderm. (B) In Brg1 KD blastocysts Oct4 (green) is widely expressed in both the ICM and cdx2-positive (red) trophectoderm. Double Oct4 & Cdx2 positive cells are shown in yellow. Blastocysts were counterstained with DAPI to visualize nuclei.
(2.26 MB TIF)

Figure S2 Phenotypic analysis of Brg1 KD and Cdx2 KD blastocysts. (A) Summary of preimplantation development. Results represent the average ± SEM from 3 experiments. A total of 60 control embryos, 53 Cdx2 KD embryos, and 48 Brg1 KD embryos were examined. Black bars, one-cell embryos injected with control siRNA; white bars, one-cell embryos injected with Cdx2 siRNA; gray bars, one-cell embryos injected with Brg1 siRNA. (B) Percentage of control embryos, Brg1 KD embryos and Cdx2 KD embryos hatching on day 4. (C) Micrographs of control blastocysts, Brg1 KD blastocysts, and Cdx2 KD blastocysts on days 4 and 5, and after 96hrs of outgrowth. Arrows indicate hatching embryos. Arrowheads highlight trophectoderm cells, and dotted lines indicate the boundary of trophectoderm outgrowth. (D) ICC analysis of Oct4 expression in control blastocysts, Brg1 KD blastocysts, and Cdx2 KD blastocysts. Blastocysts were co-stained with DAPI to visualize nuclei. (E) ICC and qRT-PCR analysis of Brg1 and Oct4 expresssion in Cdx2 KD blastocysts and control blastocysts. Blastocysts were co-stained with DAPI to visualize nuclei. qRT-PCR data were normalized to Ubtf (house keeping gene) and are relative to control blastocysts.
(1.93 MB TIF)

Figure S3 Real-time PCR analysis of Brg1 and Cdx2 transcripts in embryos microinjected with Brg1 and Cdx2 siRNA. (A) Microinjection of Brg1 siRNA or Brg1 siRNA and Cdx2 siRNA combined triggers a similar reduction in Brg1 transcripts in preimplantation embryos. (B) Microinjection of Cdx2 siRNA or Cdx2 siRNA and Brg1 siRNA combined induces a similar reduction in Cdx2 transcripts in preimplantation embryos. Data were normalized to Ubtf (house keeping gene) and are relative to control blastocysts. Different letters denote statistical significance (p<0.05).
(1.08 MB TIF)
