# Supplementary figures and images for: Brg1 Is Required for Cdx2-Mediated Repression of Oct4 Expression in Mouse Blastocysts
Source: PLoS One. 2010 May 12;5(5):e10622. doi: 10.1371/journal.pone.0010622 (PMC2868905; doi:10.1371/journal.pone.0010622)

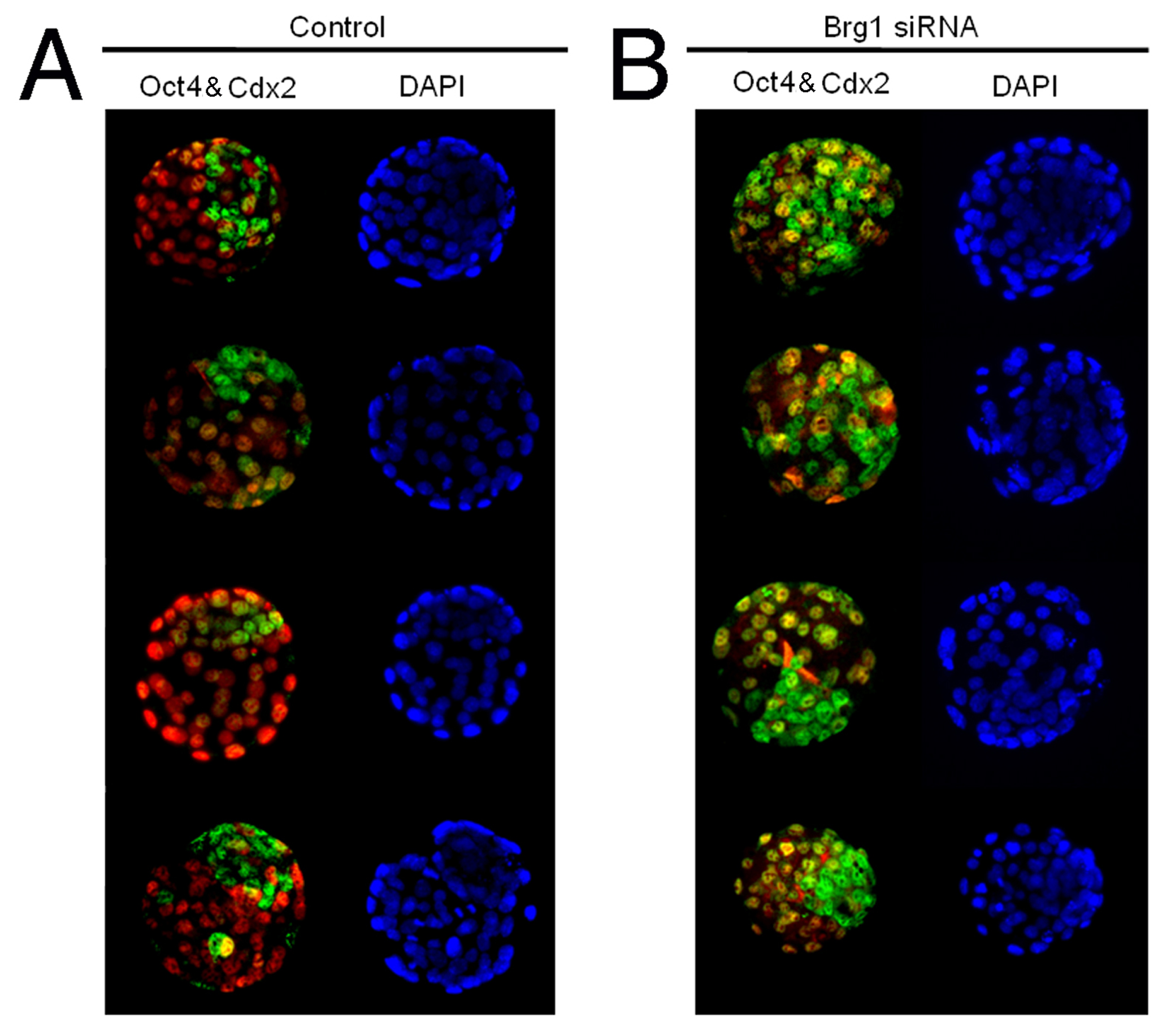

Supplement: Figure S1 — Expression and localization of Cdx2 and Oct4 in Brg1 KD and control blastocysts. (A) In control blastocysts Oct4 expression (green) is restricted to the ICM and is largely absent in the Cdx2-positive (red) trophectoderm. (B) In Brg1 KD blastocysts Oct4 (green) is widely expressed in both the ICM and cdx2-positive (red) trophectoderm. Double Oct4 & Cdx2 positive cells are shown in yellow. Blastocysts were counterstained with DAPI to visualize nuclei. (2.26 MB TIF) [file pone.0010622.s001.tif]

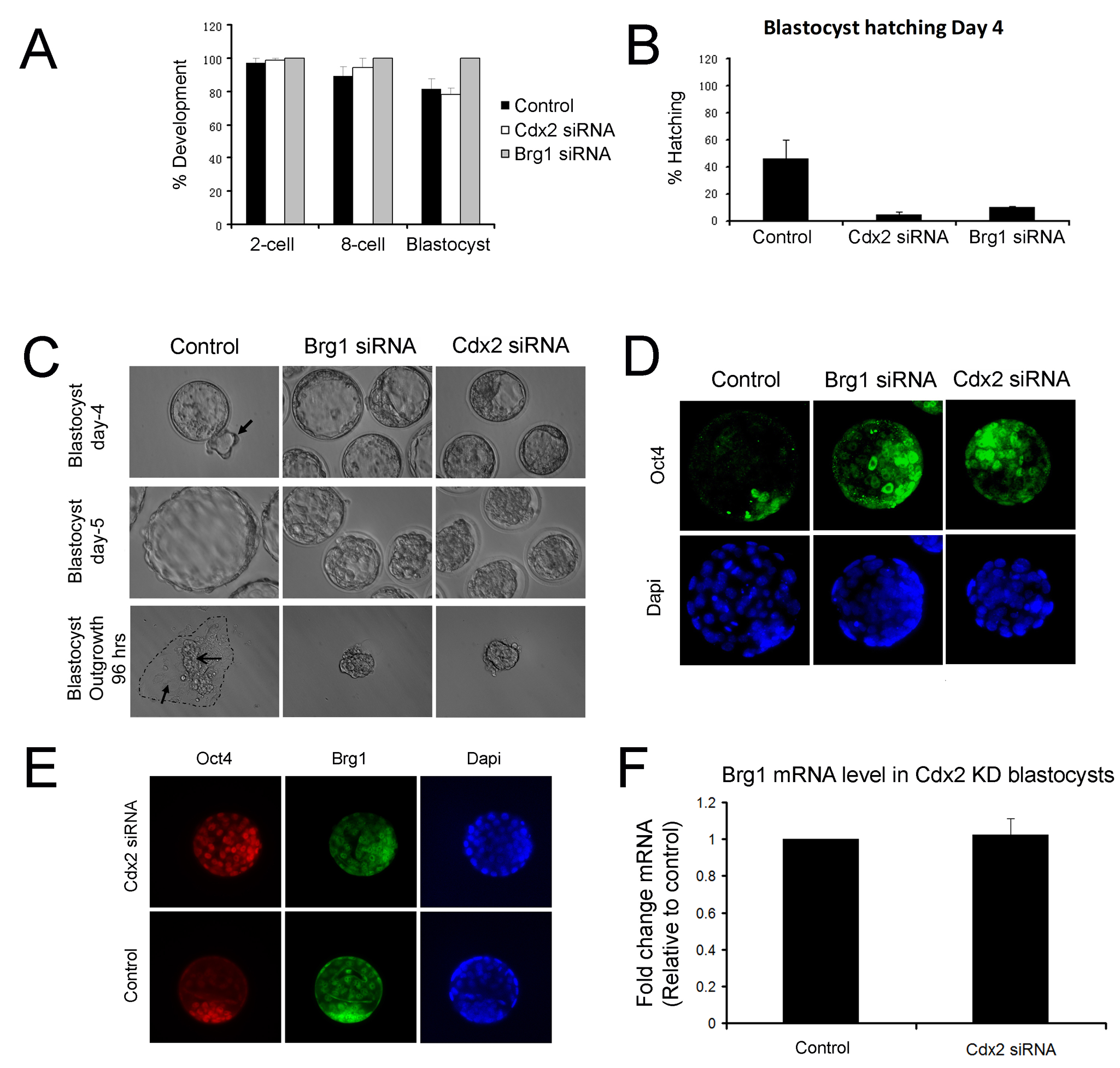

Supplement: Figure S2 — Phenotypic analysis of Brg1 KD and Cdx2 KD blastocysts. (A) Summary of preimplantation development. Results represent the average ± SEM from 3 experiments. A total of 60 control embryos, 53 Cdx2 KD embryos, and 48 Brg1 KD embryos were examined. Black bars, one-cell embryos injected with control siRNA; white bars, one-cell embryos injected with Cdx2 siRNA; gray bars, one-cell embryos injected with Brg1 siRNA. (B) Percentage of control embryos, Brg1 KD embryos and Cdx2 KD embryos hatching on day 4. (C) Micrographs of control blastocysts, Brg1 KD blastocysts, and Cdx2 KD blastocysts on days 4 and 5, and after 96hrs of outgrowth. Arrows indicate hatching embryos. Arrowheads highlight trophectoderm cells, and dotted lines indicate the boundary of trophectoderm outgrowth. (D) ICC analysis of Oct4 expression in control blastocysts, Brg1 KD blastocysts, and Cdx2 KD blastocysts. Blastocysts were co-stained with DAPI to visualize nuclei. (E) ICC and qRT-PCR analysis of Brg1 and Oct4 expresssion in Cdx2 KD blastocysts and control blastocysts. Blastocysts were co-stained with DAPI to visualize nuclei. qRT-PCR data were normalized to Ubtf (house keeping gene) and are relative to control blastocysts. (1.93 MB TIF) [file pone.0010622.s002.tif]

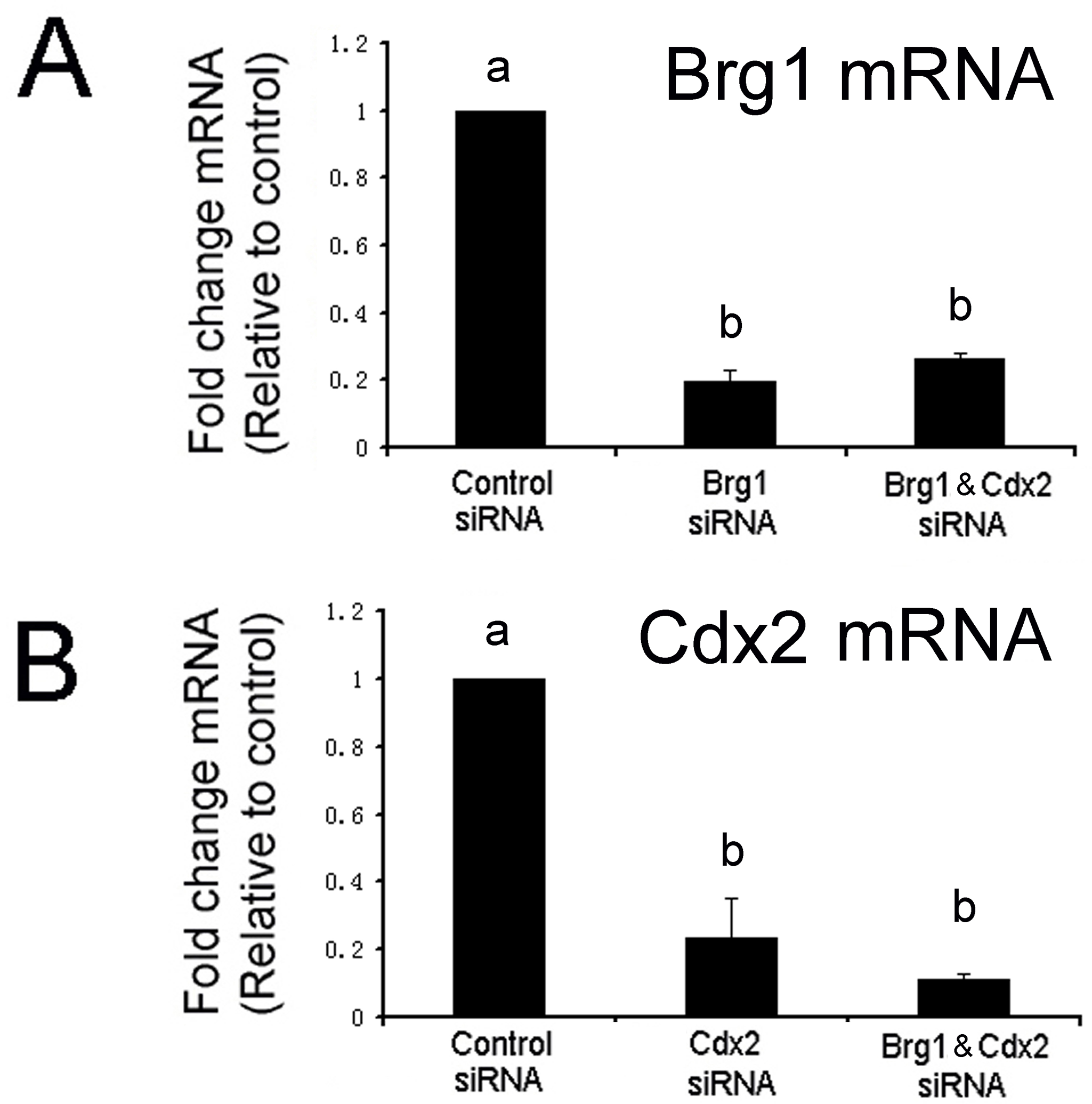

Supplement: Figure S3 — Real-time PCR analysis of Brg1 and Cdx2 transcripts in embryos microinjected with Brg1 and Cdx2 siRNA. (A) Microinjection of Brg1 siRNA or Brg1 siRNA and Cdx2 siRNA combined triggers a similar reduction in Brg1 transcripts in preimplantation embryos. (B) Microinjection of Cdx2 siRNA or Cdx2 siRNA and Brg1 siRNA combined induces a similar reduction in Cdx2 transcripts in preimplantation embryos. Data were normalized to Ubtf (house keeping gene) and are relative to control blastocysts. Different letters denote statistical significance (p<0.05). (1.08 MB TIF) [file pone.0010622.s003.tif]
